# Living donor liver transplantation for congenital absence of portal vein in portal venous reconstruction with a great saphenous vein graft

**DOI:** 10.1186/s40792-020-00916-8

**Published:** 2020-06-29

**Authors:** Peilin Li, Masaaki Hidaka, Takashi Hamada, Satoshi Ikeda, Shinichiro Ono, Yasuhiro Maruya, Tota Kugiyama, Takanobu Hara, Tomoko Yoshimoto, Tomohiko Adachi, Takayuki Tanaka, Takayuki Miyoshi, Shunsuke Murakami, Yu Huang, Kengo Kanetaka, Susumu Eguchi

**Affiliations:** 1 Department of Surgery, Nagasaki University Graduate School of Biomedical Science, 1-7-1 Sakamoto, Nagasaki, 852-8102 Japan; 2 Department of Cardiovascular Medicine, Nagasaki University Graduate School of Biomedical Science, 1-7-1 Sakamoto, Nagasaki, 852-8102 Japan

**Keywords:** Congenital absence of portal vein, Living donor liver transplantation, Portal vein restructure, Great saphenous vein transplantation

## Abstract

**Background:**

Congenital absence of portal vein (CAPV) is a rare structural anomaly in which the portal vein (PV) blood that normally flow into the liver directly drains into the systemic venous system through other collateral circulation. Congenital portal vein shunts (CPSs) is classified into types I and II according to the absence or presence of the intrahepatic portal vein, respectively. The CPS type I is also known as CAPV. The liver transplantation may be the only treatment option for CAPV. The key point of liver transplantation for CAPV is the reconstruction of the PV.

**Case presentation:**

A 29-year-old man was diagnosed with CAPV with splenomegaly and gastroesophageal varix when being treated for pancytopenia and liver dysfunction. A living donor liver transplantation was performed for him using the right lobe which had been donated by his mother. The PV was reconstructed using his own great saphenous vein (GSV) as a graft vein. The end of the GSV graft was anastomosed to the inferior mesenteric vein while the other end was anastomosed to the vein graft of the right hepatic vein from the explanted liver.

**Conclusion:**

Using the patient’s own GSV for PV reconstruction during living donor transplantation in the patient with CAPV seems to be an effective method.

## Background

Congenital absence of portal vein (CAPV) is a rare structural abnormality in intrahepatic and extrahepatic veins. It is characterized by part or all of the mesenteric blood directly draining into the systemic venous circulation through shunts, also known as Abernethy malformation [1–3]. Morgan divided congenital portal vein shunts (CPSs) into types I and II according to the absence and presence of intrahepatic portal vein, respectively [4]. Lautz et al. [5] further classified the CPSs into five types. Type I, also called CAPV, has no intrahepatic portal venous flow, while type II has partial shunt with a preserved hepatic portal flow; shunt arising from a branch of the portal vein (PV) was classified as type IIa, and shunts arising from the main PV or its bifurcation were classified as type IIb, while those arising from the mesenteric, gastric, or splenic veins were classified as type IIc. The classification and diagnosis of CPSs is made by radiological examinations, including magnetic resonance imaging (MRI) and computed tomography (CT) [6].

Liver transplantation (LT) is recommended for CAPV treatment, especially CAPV complicated with portal shunt, pulmonary hypertension, or liver nodules. However, due to the congenital absence of a PV, the selection of the PV graft is difficult during PV reconstruction, especially when the shunt stump is too short. When transplanted in situ, the use of the donor’s ovarian vein and the patient’s hepatic vein (HV) as graft veins has been reported [7, 8], but the use of the patient’s own great saphenous vein (GSV) as the graft vein for a patient with CAPV has never been described, although the GSV was reportedly used as graft in living donor liver transplantation (LDLT) for other diseases [9, 10].

We herein report a case of CAPV, in which LDLT was performed for the patient and the patient’s own GSV was used to reconstruct the PV.

## Case report

This case was diagnosed to have CAPV with splenomegaly and gastroesophageal varix while being treated for pancytopenia (low white blood cell and platelet count) and liver dysfunction at 13 years of age in 2003. In 2016, he suffered cardiopulmonary arrest due to pulmonary hypertension. He failed to receive LDLT because of uncontrolled pulmonary hypertension. In 2018, he visited our hospital for a LDLT assessment.

**Fig. 1:**
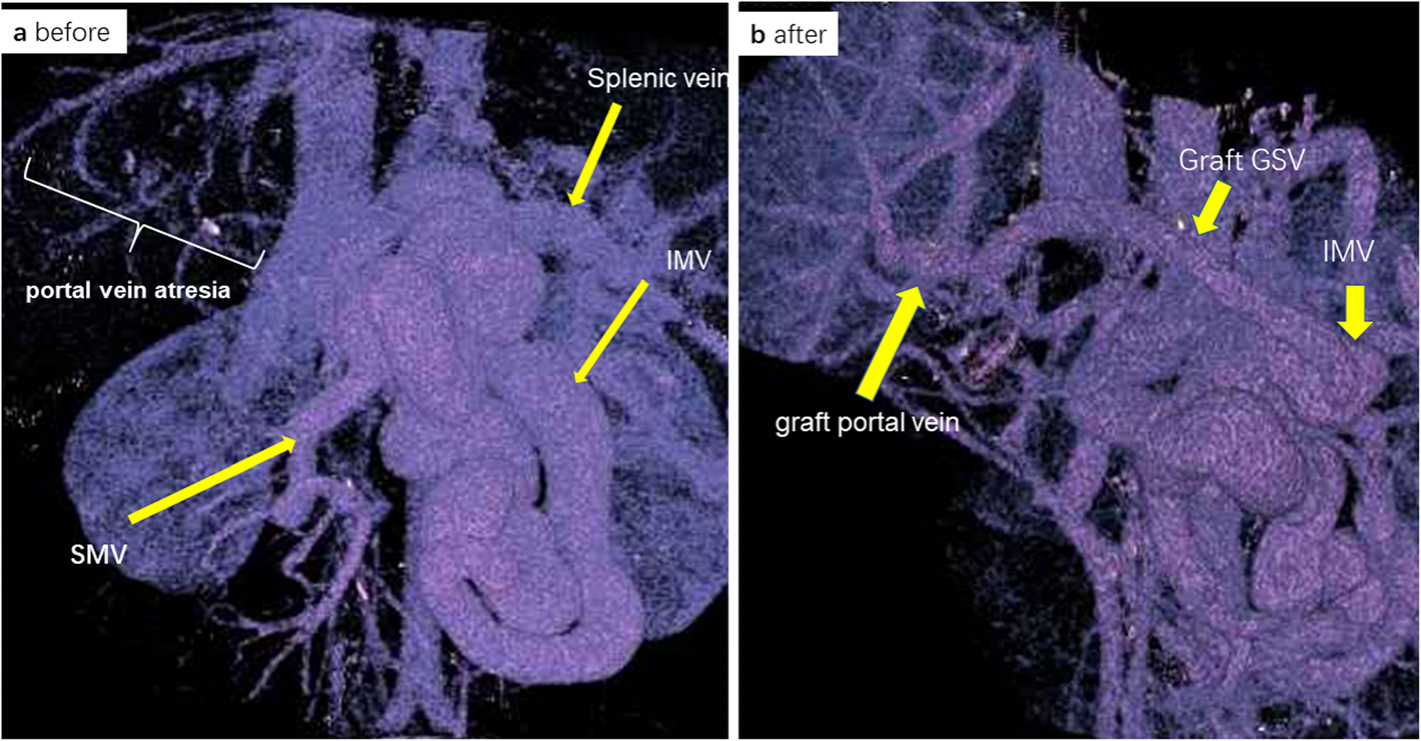
Three-dimensional reconstruction images of the PV system before and after LDLT. **a** Preoperative image showed PV atresia with an enlarged IMV from splenic vein to IVC. **b** Postoperative image showed the patency of the PV and the venous graft GSV at 2 to 3 months after LDLT

A routine examination on admission showed the patient’s liver function Child-Pugh score to be 8 (B), and his MELD score was 15. Upper gastrointestinal endoscopy findings were consistent with signs of esophageal varices. Radiological three-dimensional reconstruction showed PV atresia. The enlarged inferior mesenteric vein (IMV) was shunted through the splenic vein to the inferior vena cava (IVC), indicating that the patient was CPSs type I (CAPV) (Fig. 1a). Hyperplasia nodules could be seen in the liver, suggesting a benign lesion. We performed LDLT with a right posterior graft from his mother. Right heart catheterization in October 2018 showed a mean pulmonary arterial pressure (mPAP) of 36 mmHg, a cardiac output of 9.37 L/min (determined by the Fick method), and a pulmonary vascular resistance (PVR) of 2.77 Wood units. Prior to the liver transplantation, his pulmonary hypertension had been treated with continuous venous infusion of treprostinil (maximum dose, 126 ng/kg/min), the oral administration of riociguat (2.5 mg, T.I.D.), and the inhalation of iloprost (10 μg, Q.I.D.). The mean pulmonary artery pressure was controlled to between 19 and 24 mmHg. We assessed that good control of pulmonary hypertension had been achieved and that the patient had a good right cardiac function and was eligible for living donor liver transplantation.

**Fig. 2:**
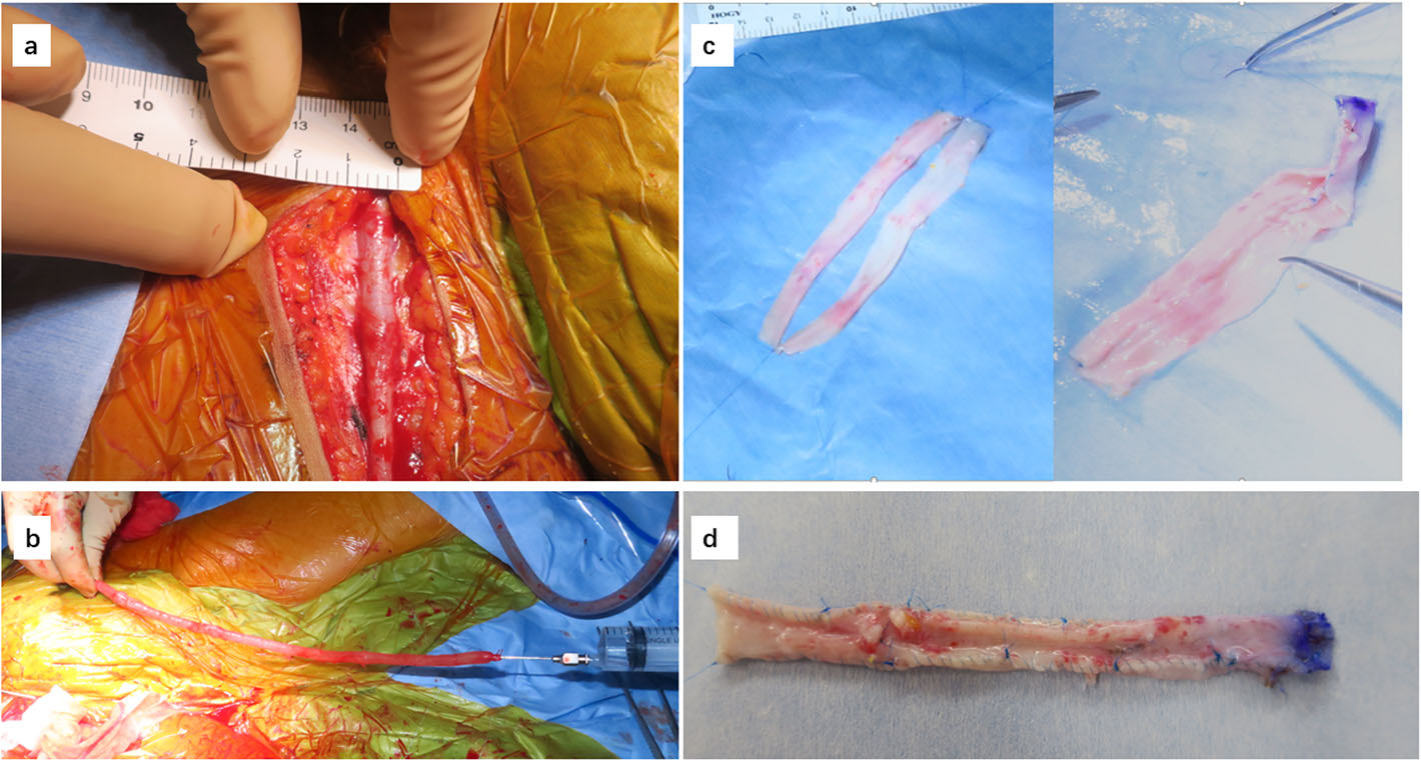
The GSV preparation. **a** The diameter of the GSV was ensured to be about 1 cm. **b** The length was about 33 cm. **c, d** The GSV was divided into two parts, and then each part was cut longitudinally. They were stitched into a tube shape

For this patient, reconstruction of the PV was the key point. The surgical procedures were as follows: first, the right GSV (1 cm in diameter and 33 cm in length) was obtained by a cardiovascular surgeon (Fig. 2a). The right GSV was then injected with heparin sodium solution to ensure no leakage (Fig. 2b). The GSV was divided into two parts, and then each part was cut longitudinally and stitched into a tube shape which was used as the PV graft later (Fig. 2c, d). During the liver resection, we confirmed and cut the recipient’s hepatic arteries and bile ducts, but an obliterated PV was identified during surgery. After liver resection, we opened the omentum and identified the IMV below the pancreas.

**Fig. 3:**
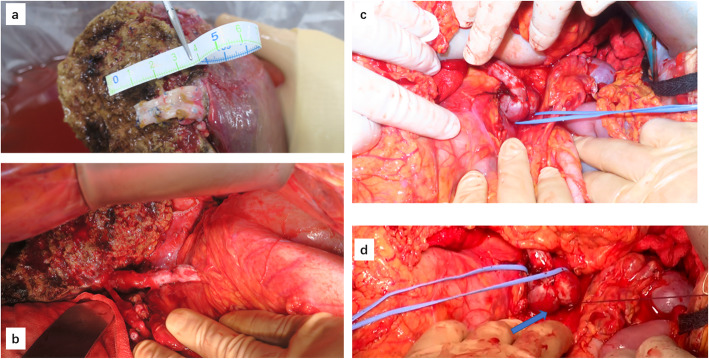
The PV reconstruction. **a** The posterior branch of the PV was anastomosed with a vein graft of the right HV from the explanted liver because of the short cuff of the PV. **b** The patient’s tubular graft GSV was anastomosed to the IMV. **c** The patient’s graft GSV was anastomosed to a vein graft of the right HV. **d** We performed ligation with string at the shunt from the splenic vein to the renal vein

On back table, we prepared PV reconstruction using the recipient’s right HV from the explanted liver because of its short cuff (Fig. 3a). After fixing the IMV with Satinsky forceps, the IMV was anastomosed with the tubular GSV using 5-0 Surgipro sutures (Fig. 3b). After venous anastomosis, PV reconstruction was performed, and the graft GSV was anastomosed with the recipient’s right HV graft using 5-0 Surgipro sutures (Fig. 3c). After reperfusion of the graft, ultrasound showed good blood flow of the PV. We then ligated the IMV shunt vein at the shunt from the splenic vein to the renal vein to prevent blood steal (Fig. 3d). Finally, we finished the operation after reconstructing the hepatic artery and bile duct.

Postoperative treatment for pulmonary hypertension was managed with treprostinil (iv), sildenafil (60 mg 3× [2 days]) nitric oxide inhalation (max 20 ppm), and olprinon (a PDE-III inhibitor). Regarding the post-operative course, since the pulmonary artery pressure was slightly high at the beginning, the amount of treprostinil was initially increased, but there was also a decrease in the systemic blood pressure. Since it did not increase, it was discontinued at 22 days after surgery. NO inhalation was initiated on the day after surgery, but was discontinued after 20 days due to improvement. The patient underwent emergency surgery due to early postoperative bleeding. However, 3 months later, he died of sepsis, despite recovering from the postoperative bleeding and hemodynamic complications. Three-dimensional reconstruction had shown the patency of PV and venous graft GSV 2 to 3 months after LDLT (Fig. 1b).

## Discussion

CAPV is a rare congenital portal shunt without hepatic PV perfusion that was first reported in 1793 by John Abernethy [1]. Morgan divided CPSs into types I and II according to the presence or absence of the intrahepatic portal vein, respectively, and defined liver perfused without portal blood-total shunt as CPSs type I (CAPV) and the liver perfused with portal blood-partial shunt as CPSs type II [4]. CAPV is more common in women than in men, and it tends to be incidentally discovered more frequently in childhood than in adult. However, some patients manage to survive to adulthood.

Their liver function abnormalities cannot be detected during a physical examination. Long-term complications include benign hyperplasia, hemangioma, and primary liver cancer. The manifestations include a variety of cardiovascular system abnormalities [11–13], hepatic focal nodular hyperplasia [14], hyperbilirubinemia [15], hyperammonemia [16], mental retardation [17], and hepatic encephalopathy. For all CAPV cases, conservative treatment should be considered first, and invasive treatment, mainly portal reconstruction, can be performed when conservative treatment fail. For CAPV, invasive treatment is impossible. Liver transplantation is the only treatment. Liver transplantation was performed in the patient with CAPV, and the key point of the operation was the reconstruction of the PV [18].

Shinkai et al. [16] performed LDLT on a CAPV patient, ligating and dividing the shunt vessels, and then the stump was directly anastomated with the PV of the transplantated liver. When the stump is insufficient, venous grafts should be considered for PV reconstruction. Sumida et al. [7] performed reconstruction of the PV using a donor’s ovarian vein in LDLT for a child with CAPV. Sanada et al. [8] reported two cases of CAPV and performed reconstruction of the PV from the donor distal splenic vein or from a patent round ligament of the liver. Chen et al. [10] reported a case of PV reconstruction using the patient’s own GSV in LDLT for a patient with end-stage cirrhosis, as the patient had PV stenosis and hepatocellular carcinoma. These previous findings indicate that the GSV is indeed an option as a graft for PV reconstruction.

In the present case, we decided to use a venous graft for PV reconstruction. This patient’s GSV was used to reconstruct the PV. After removing sufficiently long and wide portion of the GSV, it was trimmed to fit the size of the PV of the graft liver. The GSV was anastomosed with the PV of the donor liver, and the other end was anastomosed with the enlarged IMV. Successful reconstruction of the PV in LDLT using the patient’s own GSV provided an effective method for managing a patient with CAPV. However, the patient experienced respiratory bleeding and atelectasis complicated with severe sepsis which directly caused his death. Therefore, pulmonary hypertension should be controlled before and after the operation, as these patients may be suffering from severe cardiovascular disease. The prevention and treatment of postoperative infection has always been a serious issue. Although CAPV is often accompanied by pulmonary hypertension and possible right heart dysfunction, with effective perioperative preparation, liver transplantation remains the preferred treatment option for the patients with CAPV. Complications after liver transplantation, including early liver dysfunction or primary liver dysfunction, postoperative complications, and severe systemic infections, remain the main causes of treatment failure, as they are in other liver transplant patients. Regarding this case, the liver graft survived, and there was no portal vein thrombosis, proving that the use of GSV grafts is an option for reconstruction of the PV in patients with CAPV.

## Conclusion

In conclusion, the key to LDLT in patients with CAPV is PV reconstruction. The present findings proved that using the GSV to reconstruct the PV in CAPV patients is an effective method.

## Data Availability

Not applicable

## Abbreviations

CAPV: Congenital absence of portal vein
PV: Portal vein
GSV: Great saphenous vein
LT: Liver transplantation
LDLT: Living donor liver transplantation
IMV: Inferior mesenteric vein
CPSs: Congenital portal vein shunts
IVC: Inferior vena cava
MRI: Magnetic resonance imaging
CT: Computed tomography
HV: Hepatic vein
